# Cerebral Neurodegeneration and Cognitive Impairment in Patients on Maintenance Hemodialysis: the Role of Neuroglia and Associated Factors (Review)

**DOI:** 10.17691/stm2026.18.1.05

**Published:** 2026-02-27

**Authors:** A.E. Khrulev, O.M. Chernova, V.N. Grigoryeva, N.A. Shiianova, N.S. Khruleva, I.V. Mukhina

**Affiliations:** MD, DSc, Associate Professor, Professor, Department of Nervous Diseases; Acting Head of the Department of Neurology, Psychiatry, and Narcology; Privolzhsky Research Medical University, 10/1 Minin and Pozharsky Square, Nizhny Novgorod, 603005, Russia; Student; Privolzhsky Research Medical University, 10/1 Minin and Pozharsky Square, Nizhny Novgorod, 603005, Russia; MD, DSc, Professor, Head of the Department of Nervous Diseases; Privolzhsky Research Medical University, 10/1 Minin and Pozharsky Square, Nizhny Novgorod, 603005, Russia; Resident, Department of Nervous Diseases; Privolzhsky Research Medical University, 10/1 Minin and Pozharsky Square, Nizhny Novgorod, 603005, Russia; MD, PhD, Associate Professor, Department of Hospital Therapy named after V.G. Vogralic; Privolzhsky Research Medical University, 10/1 Minin and Pozharsky Square, Nizhny Novgorod, 603005, Russia; DSc, Professor, Head of the Department of Normal Physiology; Head of the Scientific Research Institute of Applied and Fundamental Medicine; Privolzhsky Research Medical University, 10/1 Minin and Pozharsky Square, Nizhny Novgorod, 603005, Russia

**Keywords:** neurodegeneration, hemodialysis, cognitive impairment, neuroglia, chronic systemic inflammation, uremic toxins, brain renin-angiotensin-aldosteron system, RAAS

## Abstract

Chronic kidney disease (CKD), which often requires maintenance hemodialysis (MHD), represents a model of accelerated aging. Therefore, understanding the mechanisms underlying cerebral neurodegeneration (CND) and cognitive impairment (CI) in patients undergoing MHD is of particular importance. Brain damage in patients with end-stage CKD is considered a multicomponent process associated with the impact of cerebrovascular, neurodegenerative, and numerous dysmetabolic factors. Special attention is given to CND mechanisms mediated by the activation of microglia and astrocytes. The high prevalence of CI in the general population and especially among patients with end-stage CKD undergoing MHD determines the need to clarify CND risk factors and biomarkers. Furthermore, it is essential to explore therapeutic targets, as well as modifiable modern strategies and technologies aimed at slowing the CND progression.

This review synthesizes data on the role of neuroglia and associated factors in the development of CND and CI in MHD patients. It presents the mechanisms and factors affecting neuroglia and involved in the pathogenesis of CND and CI. Particular focus is placed on chronic systemic inflammation, the effects of uremic toxins, and the activation of the cerebral renin-angiotensin-aldosterone system. The article studies such chronic systemic inflammatory factors associated with neuroglia as protein S100B, IL-1β, IL-6, TNF-α, and fibrinogen. Experimental and clinical data investigating the impact of uremic toxins (indoxyl sulfate, p-cresol sulfate, and imidazole propionate) on microglia and astrocytes, as well as the activation of the cerebral renin-angiotensin-aldosterone system, are analyzed. Potential further clinical research fields aimed at slowing the CND and CI progression in this patient population are highlighted.

## Introduction

Cerebral neurodegeneration (CND) is a condition characterized by progressive functional impairment, structural damage, and brain neurons death. CND underlies the subsequent development of various neurodegenerative and psychiatric diseases. CND and its progression rate can influence the development and severity of various cognitive and non-cognitive neuropsychiatric (emotional-affective, behavioral, and psychotic) disorders in patients [1, 2].

According to a meta-analysis and systematic review conducted in 2022 [3], the global prevalence of mild cognitive impairment (CI) among adults aged 50 and older exceeds 15%. It is estimated that 60% of these individuals will develop clinically significant dementia within their subsequent years of life. By 2050, a several-fold increase in the number of people suffering from dementia (up to 152 million) is projected [2, 4–6].

The development of CND and CI is particularly characteristic of individuals with chronic kidney disease (CKD), including patients with end-stage CKD undergoing maintenance hemodialysis (MHD). It is important to note that from a modern perspective, CKD is considered a “model of premature/accelerated aging” [7]. Therefore, understanding the mechanisms underlying CND and CI in this patient category is important for the general population. In the Russian Federation, the CKD prevalence is 11.35% [8], with over 47,000 patients receiving MHD [9, 10]. CI of varying severity is observed in the vast majority (up to 81% of cases) of patients undergoing MHD [11–20]. Brain damage in patients with end-stage CKD is considered a multicomponent process associated with the effects of cerebrovascular, neurodegenerative, and numerous dysmetabolic factors [11, 12, 14, 21]. To date, the problem of vascular CI development in this patient category is more studied; neurodegenerative mechanisms underlying higher mental function disorders are significantly less researched [12, 22, 23]. Scientists are currently focusing on CND mediated by the activation of microglial cells and astrocytes [24–30].

**The aim of this review** is to analyze and synthesize current information on neuroglia-associated mechanisms underlying the development and progression of CND and CI in patients receiving MHD.

## Literature search strategy

The literature search was conducted using the electronic abstract databases Scopus, Web of Science, and RSCI; the PubMed search engine using the MEDLINE and PubMed Central databases; and the Springer Link, BioMed Central, Free Medical Journals, SSRN, and Google Scholar platforms for the period from 2014 to 2025. The search was performed using the following keywords: neurodegeneration, hemodialysis, cognitive impairment, neuroglia, chronic systemic inflammation, uremic toxins, brain (cerebral) renin-angiotensin-aldosterone system (RAAS).

## Pathogenesis of neuroglia-associated mechanisms of cerebral neurodegeneration and cognitive impairment in patients on maintenance hemodialysis

Disruptions in the complex interaction between neurons and neuroglia are increasingly recognized as an important pathophysiological mechanism underlying the development of CND and CI in MHD patients. In general, two main pathways of development can be identified:

1. CND predominantly mediated by neuroglial alterations;
2. CND resulting from the direct impact of factors on brain neurons.

This article will study the possible mechanisms of CND and CI related to the first pathway (mediated by effects on neuroglia).

**Figure 1.**
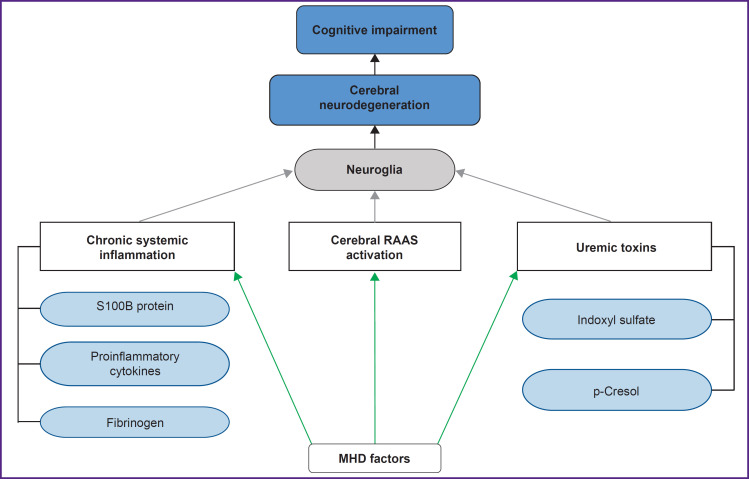
Neuroglia-associated mechanisms of cerebral neurodegeneration and cognitive impairment development in MHD patients (authors’ figure) MHD — maintenance hemodialysis; RAAS — renin-angiotensin-aldosterone system

Brain neuroglia is known to consist of macroglia (astrocytes, oligodendrocytes, ependymal cells) and microglia (predominantly mononuclear phagocytes) [24, 31, 32]. Neuroglial cells play a key role in maintaining homeostasis and regulating the brain immune responses [33, 34]. It is important to note that, to date, among all neuroglial structures, astrocytes and microglia are considered to play the primary role in the pathogenesis of CND [25, 26, 28]. The results of our analysis of neuroglia-associated mechanisms of CND and CI in MHD patients are shown in Figure 1.

Upon detection of a signal associated with physiological or pathological processes, neuroglial cells are activated and transformed, initiating various response reactions (phagocytosis, secretion of inflammatory and trophic factors) [33, 34]. It leads to neuronal proliferation and differentiation influencing synaptic connections and, therefore, cognitive and behavioral functions [33, 34].

In a healthy brain, microglia, represented by the brain resident immune cells, is believed to be in a resting state (M0) [35, 36]. When specific stressors and triggers appear, such as DAMPs (damage-associated molecular patterns) or PAMPs (pathogen-associated molecular patterns), it causes brain microgliosis. This process is implemented through PRRs (pattern recognition receptors), which play a central role in regulating innate immunity [37–39]. In this context, microglial cells can polarize from the resting state (M0) into either a proinflammatory (M1) or an anti-inflammatory (M2) phenotype [26, 30, 32, 35, 36, 39, 40]. It is believed that the specificity of microglial activation and the direction of polarization are determined by the type of damaging agent, the characteristics of the effect, and the localization of the pathological process [36].

In MHD patients, the number of factors specific to CKD and associated with dialysis that can bind with PRRs and trigger microgliosis increases. M1 microglial activation, by inducing inflammatory responses through the production of proinflammatory cytokines, contributes to neuronal damage and, thus, to neurodegeneration [36, 39, 41]. Along with the polarization of microglia into the M1 phenotype and the neuroinflammation activation during microgliosis, there occur autophagy dysregulation and CND exacerbation.

Alongside the proinflammatory microglial phenotype (M1), an anti-inflammatory phenotype (M2) is distinguished. Microglia polarization into the M2 phenotype has a neuroprotective effect as it suppresses neuroinflammation through the production of anti-inflammatory factors, leading to the restoration of a number of homeostatic processes in the brain [35, 39, 41].

Similar to microglia, the differentiation of astrocytes, being the most numerous type of macroglial cells, is also commonly subdivided into proinflammatory (A1) and anti-inflammatory (A2) [25, 27]. It is thought that in response to acute or chronic cellular distress, differentiation of astrocytes into the proinflammatory (A1) phenotype (reactive astrogliosis) becomes predominant. On the contrary, astrocyte polarization (astrogliosis) into the anti-inflammatory (A2) phenotype leads to neuroprotective and neurotrophic effects and supports the homeostasis of the blood-brain barrier (BBB) [25, 27].

MHD creates conditions for the excessive polarization of astrocytes into the A1 phenotype in the brain, which can exacerbate CND through the additional expression of neurotoxic and proinflammatory cytokines [25, 27, 42]. Furthermore, due to reactive astrogliosis, polarized A1-astrocytes lose a range of protective physiological functions that support normal neuronal homeostasis. Consequently, processes related to glutamate reuptake, glucose and fatty acid metabolism are aggravated, and synaptic impulse transmission deteriorates [25, 27, 42].

It is important to note that hemodialysis is based on the transfer of substances from the blood into the dialysate across a semipermeable membrane. Modern methods and MHD regimens allow for more effective removal of low-molecular-weight blood components, toxic substances, and metabolic products [43–47]. However, it is not possible to fully recreate the natural renal exchange with an “artificial kidney” machine. Some of the metabolic products remaining in the blood can cross the BBB and alter neuroglial activity. Additionally, during hemodialysis, it is not possible to cleanse the blood of substances with a molecular weight exceeding 30,000 Da, as these molecules cannot pass through modern dialysis membranes [44, 45]. It appears that the CND rate and the degree of proinflammatory polarization of neuroglia also depend on the hemodialysis technologies (hemodialysis, hemofiltration, or hemodiafiltration) and the properties of the dialysis membrane [43–46].

In general, the role of microglia and astrocytes in the CND development in CKD patients undergoing MHD remains unclear. The molecular pathway determining the direction of phenotypic differentiation of microglia/ astrocytes from M0/A0 to M1/A1 or M2/A2 is currently not fully understood. The definitive role of morphological and functional cellular polarization in CND in the general population, as well as in MHD patients, also requires clarification. Possible mechanisms of neuroglial activation mediated by the interaction of CKD-specific and dialysis-associated factors with cytokine receptors will be presented further below. Currently, there is no data on the hemodialysis influence on other receptor-dependent mechanisms of neuroglial regulation.

Thus, the neuroglia in MHD patients is under unfavorable conditions that promote microglial activation, reactive astrogliosis, and CND [44–46, 48]. Based on available experimental and clinical data, chronic systemic inflammation, persistent exposure to uremic toxins, and activation of the cerebral RAAS are classified as neuroglia-associated mechanisms underlying CND and CI and their exacerbation in MHD patients. These will be discussed in more detail further below.

## Chronic systemic inflammation as a factor in the cerebral neurodegeneration development in patients on maintenance hemodialysis

According to a number of clinical studies, patients with end-stage CKD and, in particular, those receiving MHD have signs of chronic systemic inflammation [16, 49–52]. This condition is considered to result in an increased risk of cardiovascular events, the malnutrition-inflammation complex syndrome (MICS), anemia, mineral and bone disorders, as well as CND and CI [21, 50, 53]. Research provides data on factors of chronic systemic inflammation that affect neuroglia in MHD patients, such as protein S100B, some proinflammatory cytokines, and fibrinogen [21, 50, 53, 54].

### Protein S100B

**Figure 2.**
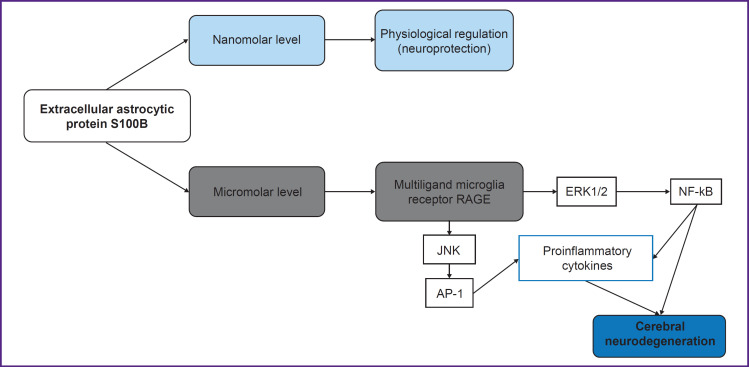
Mechanisms of cerebral neurodegeneration mediated by higher (micromolar) concentrations of extracellular astrocytic S100B (adapted from [59, 60]) The binding of extracellular astrocytic protein S100B with receptors initiates various signaling cascades in target cells: at low (nanomolar) levels, extracellular S100B leads to physiological cellular regulation and does not affect microglial homeostasis; at higher (micromolar) levels, it leads to cerebral neurodegeneration [55–58]. RAGE — receptor of advanced glycation end-products; ERK1/2 — extracellular signal-regulated kinase 1/2; NF-κB — nuclear factor kappa B; JNK — c-Jun N-terminal kinase; AP-1 — activator protein 1

Protein S100B (molecular weight 21 kDa) is a calcium-binding protein of the S100 family, concentrated in the central nervous system primarily within astrocytes [21, 55–57]. Several authors [55, 57, 58] classify S100B as a marker of brain damage. At the same time, Michetti et al. [57] suggest that extracellular S100B protein itself may not only be a damage biomarker but also an independent pathogenetic factor provoking neuroinflammation and CND. The CND development amid pathologically elevated (micromolar) concentrations of extracellular S100B protein occurs through the activation of intracellular pathways (intracellular calcium overload, accumulation of reactive oxygen species), the release of proinflammatory cytokines by microglia, and the activation of neuronal apoptosis (Figure 2) [55–60].

Fomina et al. [59] studied the correlation between cognitive functions and S100B levels in young and middle-aged individuals with CKD stages I–III (n=108). CI was determined using the Mini-Mental State Examination (MMSE). Elevated S100B concentrations were found in the early CKD stages and increased as the glomerular filtration rate decreased (rs=–0.37, p=0.001). Elevated concentrations were associated with neurodynamic CI, in particular, with the minimum and average exposure in complex visual-motor response (rs=0.29, p=0.001; rs=0.39, p=0.001, respectively), as well as with the total anticipation time and the number of accurate reactions in tests with moving objects (rs=–0.39, p=0.001; rs=–0.31, p=0.001).

Park et al. [15] provide data on the correlation between CI and increased S100B concentration in patients with end-stage CKD. The study involved 30 patients receiving MHD three times a week (session duration not specified), with a mean dialysis duration of 21 months. The cognitive function of study participants was assessed using the MMSE scale. Patients were divided into two groups: without CI (n=17) and with CI (n=13). Multiple logistic regression analysis showed that the serum S100B concentration in patients with CI was significantly higher and was an independent predictor of CI development (S100B≥36.1 pg/ml). The authors link these results to changes in brain neuroglia in patients on MHD and the high content of extracellular astrocytic S100B. It should be mentioned that the conducted study had some limitations: a relatively small sample size of the participants and the screening nature of the chosen neuropsychological assessment method using the MMSE, which may have affected the reliability of the results.

The data obtained by Park et al. [15] are consistent with the results of later studies. For example, Al-Hakeim et al. [54] studied the association of a number of blood inflammatory biomarkers, including S100B, with the development of neuropsychiatric symptoms in patients receiving MHD (n=70). Elevated S100B concentrations were associated with the development and severity of one or more neuropsychiatric syndromes. Bossola et al. [60], apart from analyzing S100B levels before or after a hemodialysis session, also investigated the association of “post-dialysis fatigue” with the S100B serum concentration. A total of 30 patients on MHD participated in the study. Twenty-two of them (73.4%) had the “post-dialysis fatigue” syndrome. The serum S100B concentration after dialysis (median [interquartile range] = 17.4 μg [7.1–30.9]) was significantly higher than the corresponding pre-dialysis level (median [interquartile range] = 5 μg [1.4–22.1]; p=0.0001) [60].

In general, it should be noted that there are not enough publications focusing on the extracellular S100B level and its role in the development of CND and CI in patients receiving MHD [15, 54, 60]. Definitive role of S100B in CND development is not fully clear, further clinical research is necessary. This indicator of chronic systemic inflammation may potentially be proposed as a biomarker of the CND severity. Moreover, the use of S100B inhibitors capable of crossing the BBB may prove to be a promising CI treatment in this patient category in the future [56, 61].

### Proinflammatory cytokines interleukin-1β, interleukin-6, and tumor necrosis factor alpha

Factors of chronic systemic inflammation associated with an impact on neuroglia via its cytokine receptors include numerous different pro-inflammatory cytokines [33, 62]. It should be mentioned that astrocytes and microglia express a wide range of cytokine receptors on their cell surfaces and can produce a large number of cytokines. Below are the proinflammatory cytokines that have been studied in experimental or clinical research regarding the hemodialysis effect on neuroglia. The activation of neuroglia is believed to depend on the levels and ratios of proinflammatory cytokines that have crossed the BBB and potentiate CND and CI [33, 62, 63]. Data indicate high concentrations of a number of proinflammatory cytokines in pre-dialysis and dialysis patients [64, 65]. It may be connected with the effects of CKD- and dialysis-related factors (blood interaction with the dialysis membrane, chronic circulation of uremic toxins, persistence of chronic infectious foci, translocation of gut microbiota into the bloodstream) [53, 64–67]. Particular research interest lies in the potential role of interleukins (IL-1β, IL-6) and tumor necrosis factor alpha (TNF-α) in the CND development in pre-dialysis and dialysis periods. It has been noted that elevated extracellular concentrations of these cytokines are associated with inflammatory activation of brain neuroglia [21, 39, 64, 65].

***IL-1β*** (molecular weight 18 kDa) being within a normal range plays an important role in the brain: it activates learning and memory processes, participates in neuronal signaling, synaptic plasticity, sleep regulation, induction of neurotrophic factors, and adult neurogenesis. It is important to note that under physiological conditions, IL-1 ligand levels are low, with microglia being the key source of IL-1 production in the brain [68]. When there is an infection and/or aseptic inflammation in the brain, IL-1β expression rapidly increases, and processes of cognitive dysfunction and CND, connected with neuroglial activation and stimulation of microglial and astrocytic proinflammatory responses, predominate [69–71]. Furthermore, IL-1β may indirectly lead to glutamate accumulation, glutamate excitotoxicity, and neuronal death [69, 72].

Currently, IL-1β is considered a key mediator of innate neuroinflammation; its level can be pharmacologically modulated. The largest number of studies [68, 73, 74] have been conducted using anti-IL-1β antibodies in experimental settings following traumatic brain injury. The goal of these studies was to reduce the neuroinflammation progression and slow CND. Most of them reported improvements in cognitive and motor functions in the subjects.

Anticytokine therapy using IL-1 receptor antagonists may become a pathogenetically substantiated field for further clinical research aimed at slowing the progression of CND and CI, including in MHD patients [75]. For example, in a multicenter, randomized, placebo-controlled study, Dember et al. [75] evaluated the safety, tolerability, and efficacy of an IL-1 receptor antagonist in 80 patients receiving MHD. Among them, 38 patients were assigned to the IL-1 receptor antagonist (anakinra, 100 mg three times per week) for 24 weeks; 42 participants were in the placebo group. MHD was performed three times per week (session duration not specified by the authors), with a mean duration of treatment with dialysis of 4.4 years. The IL-1 receptor antagonist reduced the C-reactive protein (CRP) (in 41% of cases vs. 6% in the placebo group) and IL-6 (in 25% of cases vs. 0% in the control group) plasma concentrations. The rates of serious adverse events and fatal outcomes were similar with anakinra and placebo. The rates of clinically significant adverse events (including infections and cytopenias) was significantly lower in the IL-1 receptor antagonist group compared to the control group (0.48 vs. 1.40 events/patient-year).

Overall, the role of IL-1 family cytokines in the pathogenesis of CND and CI in MHD patients is poorly understood [73]. The IL-1 receptor antagonists seem to slow the progression of CND and CI as they reduce levels of chronic systemic inflammation factors and directly block IL-1 receptors. Promising data on safety and possible efficacy on CRP and IL-6 concentrations necessitate further clinical research on the role of IL-1 blockers in this patient category.

***IL-6*** (molecular weight 21–28 kDa) is a pleiotropic cytokine, described as a powerful inducer and modulator of microglial activation in a wide range of pathological conditions. Under physiological conditions in a healthy brain, IL-6 is produced in low concentrations and regulates inflammatory and immunological responses, as well as has a neuroprotective effect [76]. When central nervous system homeostasis is disrupted (in acute ischemia, traumatic brain injury, neurodegenerative diseases), chronic IL-6 production increases; it “shifts” neuroglia into a proinflammatory phenotype and worsens CND [76, 77].

In a prospective controlled single-center study, Zhu et al. [65] investigated the expression of a few serum biomarkers in MHD patients with mild CI. The study included 58 patients undergoing MHD twice a week and hemodiafiltration once a week. The duration of one session was 240 min, with a mean dialysis duration of 6 years. The control group consisted of 20 individuals. Cognitive functions were assessed using the MMSE and the Montreal Cognitive Assessment (MoCA). In MHD patients with CI, concentrations of IL-6 and TNF-α were significantly higher. High IL-6 levels were associated with lower MoCA scores (r=0.02, p<0.0001) [65].

Monoclonal antibodies targeting the IL-6 are in advanced development to manage cardiovascular risk and reducing “vascular” inflammation in MHD patients [78, 79]. For example, a randomized phase 2b trial was completed in 2024 [79]. It studied the effect of clazakizumab, a high-affinity humanized monoclonal antibody targeting the IL-6 ligand and inhibiting downstream IL-6 function. Clazakizumab treatment (the study’s primary endpoint) significantly reduced serum high-sensitivity CRP concentrations at week 12 by 86, 90, and 92% relative to placebo in patients randomized to 2.5, 5, or 10 mg clazakizumab, respectively (p<0.0001). As for the secondary endpoints, clazakizumab treatment reduced concentrations of serum fibrinogen, amyloid A, secretory phospholipase A2, and lipoprotein(a), as well as increased mean serum albumin concentrations at 12 weeks, relative to placebo. The proportion of patients who achieved high-sensitivity CRP<2.0 mg/L was 79, 82, and 79% in the 2.5, 5, and 10 mg clazakizumab groups, respectively, compared with placebo-treated patients (0%). The study results indicate that in MHD patients, clazakizumab reduced inflammatory biomarkers associated with cardiovascular events. Patients’ cognitive functions and neuroglial status were not studied.

***TNF-α*** (molecular weight 17 kDa) is a multifunctional molecule also involved in neuroinflammation [80, 81]. Most researchers emphasize its role in CND and CI, mediated by neuroglia activation and neurotoxicity enhancement [39, 80, 81]. TNF-α is hypothesized to enhance M1 activity but reduce the activity of M2 markers in microglia; it initiates autocrine activation and glutamate excitotoxicity and oxidative stress; it also disrupts insulin resistance and autophagy in brain cells [39, 81]. Data indicate that TNF-α-induced neurotoxicity was alleviated with rapamycin pretreatment of microglia [39].

Several clinical studies demonstrated elevated TNF-α plasma concentrations in MHD patients [51, 82]. Elevated serum TNF-α concentration was shown to be associated with CI in dialysis patients [65]. Furthermore, in the aforementioned study by Zhu et al. [65], aside from IL-6, the correlation between serum TNF-α levels and CI in MHD patients was analyzed. It was found that in patients with mild CI, a high TNF-α level was associated with a lower MoCA score used to assess neuropsychological status (r=0.27, p<0.0001).

Oral bioactive supplements may become another promising clinical research direction aimed at normalizing levels of proinflammatory cytokines in MHD patients and slowing the progression of CND and CI [51, 67]. Vafadar-Afshar et al. [67] showed that, in 54 MHD patients, serum levels of IL-6 and TNF-α decreased after 12 weeks of treatment with nanocurcumin compared to placebo (p=0.024 for IL-6 and p=0.02 for TNF-α). The expression of *IL-6* and *TNF-α* genes in peripheral blood mononuclear cells was also reduced at 12 weeks of treatment compared to baseline, in the nanocurcumin group. Changes in gene expression correlated with changes in serum levels of IL-6 and TNF-α. Supriyadi et al. [51] studied the effect of superoxide dismutase (SOD) on TNF-α levels in MHD patients [51]. The study included 28 patients (mean age 42±11 years) receiving MHD twice a week (session duration not specified), with a mean dialysis duration of 24 (5–72) months. All participants received SOD-gliadin 250 IU twice a day for 4 weeks. Serum TNF-α levels were assessed before and after treatment. Exogenous supplementation with SOD-gliadin led to a decrease in plasma TNF-α level from 0.109 (0.087–0.223) to 0.099 (0.083–0.149) pg/ml (p=0.036).

The sample size was relatively small, indicating the need for further controlled clinical trials.

### Fibrinogen

Fibrinogen (molecular weight 340 kDa) is a glycoprotein of the blood coagulation system that acts as the precursor for fibrin clot. It is synthesized in the liver and circulates in the blood plasma [48, 83, 84]. Since high fibrinogen levels are associated with an increased risk of dementia, researchers attribute a significant role to fibrinogen in the development of CND [83–86]. BBB breakdown allows fibrinogen to leak from the blood into the brain, where it is converted into insoluble fibrin by perivascular tissue factor and thrombin and deposited in the brain parenchyma and vessels. Thrombin-mediated fibrinogen cleavage exposes the P2 epitope, which can bind CD11b and CD11c on neuroglia. Consequently, fibrin being in brain triggers neurotoxic polarization of microglia and astrocytes, contributing to CND [83–88]. To date, it has been demonstrated that plasma fibrinogen levels correlate with plasma levels of beta-amyloid Aβ40, Aβ42, and phosphorylated tau-181 (p-tau) in cerebrospinal fluid, as well as with indicators of Aβ deposition in the brain (such as p-tau/Aβ42) and in the cerebral blood vessel wall [88].

Pyun et al. [89] investigated the cognitive profile according to plasma fibrinogen levels. This retrospective study included 643 patients with mild CI. 323 patients had high fibrinogen levels and 320 patients had low fibrinogen levels. Cognitive functions were assessed using the MMSE scale, the Clinical Dementia Rating Scale Sum of Boxes (CDR-SOB), and the Global Deterioration Scale (GDS). Patients with mild CI and higher plasma fibrinogen levels demonstrated poorer performance in attention and executive function, independent of apolipoprotein E genotype or other vascular risk factors. The authors emphasize that one mechanism for CI development in patients with high fibrinogen levels is CND mediated by neuroglial activation. The findings of Pyun et al. [89] are consistent with several other clinical studies that also demonstrated a correlation between high fibrinogen levels and low performance across various cognitive functions (memory, attention, information processing speed, and executive functions) [90–92].

Blood fibrinogen level is considered elevated in MHD patients [48, 83]. For instance, Pénzes et al. [48] studied serum fibrinogen concentration in patients receiving MHD before and after a 4-h hemodialysis course. The study involved 30 patients; 240-min MHD sessions were provided three times a week, with a mean dialysis duration of 4.5 years. More than 50% of MHD patients had elevated fibrinogen concentrations above the reference range (1.5–4.0 g/L); the remaining values were mostly in the upper half of the reference range. The mean fibrinogen concentration was 4.21±0.82 g/L in patients treated with hemodiafiltration and 4.23±0.87 g/L in the hemodialysis group [48]. Following a 4-hour hemodialysis, plasma fibrinogen concentration increased. According to the authors, high fibrinogen levels in MHD patient are connected with chronic hemostatic alterations and elevated CRP levels due to chronic systemic inflammation. They consider CRP level, characterizing the inflammatory status, to be the key factor determining fibrinogen concentration.

It is important to note that studies specifically investigating CND and CI in MHD patients in relation to their fibrinogen levels and BBB status are currently lacking.

## The role of uremic toxins in cerebral neurodegeneration and cognitive impairment in patients undergoing maintenance hemodialysis

In addition to factors related to chronic systemic inflammation, modern data show the significant role of uremic toxins in CND and CI in MHD patients [16, 17, 93, 94]. Amino acids present in dietary protein serve as fermentation substrate for bacteria in the large intestine, particularly when consumed in excess [95]. Uremic toxins are residues of organic intestinal compounds, bacterial amino acid metabolites. They cannot be eliminated from the body due to impaired kidney function and consequently accumulate in the blood and brain [16, 96, 97]. Excessive and prolonged exposure to uremic toxins is considered a cause of neuroglial activation, as well as their negative effects on brain neurons and CND [16, 28, 93, 98]. Researchers suggest that common pathophysiological aspects of these processes include reduced synaptogenesis rate, decreased sodium-potassium ATPase activity, neurotransmitter imbalance, and increased BBB permeability [16, 93, 99–102]. The entry of uremic toxins into the brain is mediated by specific transporters (organic anion transporters) [16, 93, 100]. A potential role has been demonstrated for indoxyl sulfate (IS), p-cresol and its derivative p-cresol sulfate (PCS), as well as imidazole propionate, in activating neuroglia in MHD patients. It is important to note that these molecules are difficult to eliminate via dialysis due to their high plasma protein binding ability [93, 95].

***Indoxyl sulfate*** (IS; molecular weight 213 Da) is a tryptophan metabolite [103–105]. This uremic toxin begins to accumulate in patients at the pre-dialysis CKD stages and is poorly eliminated via hemodialysis [93, 104, 106]. IS may trigger CND as it negatively affects microglial and astrocyte function through the induction of oxidative stress, proinflammatory cytokine production, and apoptosis in neuroglial cells [23, 28, 38, 107].

The controlled study by Lin et al. [93] showed an association between elevated blood free IS levels and CI in 260 patients (mean age 58.1±11.3 years) receiving MHD. Hemodialysis was performed three times a week, with each session lasting 240 min and a mean dialysis duration of 7 years. The dialysate flow was maintained at 500 ml/min, the blood flow rate — at 250 and 300 ml/min, and the dialysis adequacy index Kt/V was more than 1.2. Circulating free form IS and PCS were measured using tandem mass spectrometry. Patient cognitive function was evaluated using the MMSE and the Cognitive Abilities Screening Instrument (CASI). The ability of IS and PCS to cross the BBB was predicted using *in silico* computer modeling. According to the assessment, both compounds were classified as BBB+ and had a high probability of BBB penetration (0.8727 for IS and 0.9405 for PCS). MHD patients had CI and higher circulating IS and PCS levels more frequently compared to the control group. An association was found between high plasma IS level (>1.45 μg/ml) and low scores on cognitive tests (long-term memory, abstract thinking, language, and spatial construction). The authors conclude that a high level of circulating free form IS, but not PCS is associated with CI, which is likely due to the increased protein binding ability of IS and its poorer elimination during dialysis compared to PCS [93].

Further clinical studies are needed to evaluate the effect of IS elimination and its reduced production on slowing down CND and CI in MHD patients.

***p-Cresol*** (molecular weight 108 Da) being a tyrosine metabolite is another intestinal neurotoxin that induces apoptosis, oxidative stress, and neuroinflammation. p-Cresol and PCS accumulate in the blood of MHD patients and, at high concentrations, negatively affect neuroglia [95, 96, 99, 105, 106, 108]. Given that the gut microbiota is the primary source of endogenous p-cresol, the balance among gut microbiota strains (particularly *Clostridium* species) may directly influence neuroplasticity and CND [49, 109].

Experiments showed an association between high p-cresol concentration and behavioral disorders in animals. For instance, in a study by Sun et al. [99], unilateral nephrectomized (n=68) and intact control (n=20) mice received PCS intraperitoneally at a dose of 100 mg/kg for 7 weeks. PCS administration increased its concentration in prefrontal cortex tissues, while the administration of a uremic toxin adsorbent reduced it. The following behavioral tests were conducted to assess changes: open field test, Morris water maze, forced swim test, tail suspension test, and light/dark box test. Following PCS exposure, unilateral nephrectomized mice had PCS accumulated in the brain and they developed depression-like and/or anxiety-like behavior compared to the control group. Elevated p-cresol levels were found to increase the expression and activity of a range of molecules (IL-1β, JNK, p38, NF-kB, AP-1) in prefrontal cortex tissues. Furthermore, PCS administration led to decreased concentrations of BDNF and serotonin. The authors identified an association between high and low concentrations of the aforementioned uremic toxin molecules, the survival of prefrontal cortex neurons, neurogenesis, and neuropsychological assessment results.

Thus, the data indicate that brain neuroglial cells are targets of the negative effects of uremic toxins [93, 99]. However, publications dedicated to the role of IS and PCS in CND and CI in MHD patients are insufficient. In the future, IS and PCS may become therapeutic targets for neurodegenerative diseases in CKD patients. Consequently, the development and application of uremic toxin adsorbents may be a promising scientific and practical field for slowing the progression of CND and CI in this patient category [97, 105, 110, 111]. However, testing this scientific hypothesis requires further clinical studies.

## Activation of the cerebral renin-angiotensin-aldosterone system as a factor in the development of cerebral neurodegeneration and cognitive impairment in patients undergoing maintenance hemodialysis

The RAAS is an endocrine system widely recognized for its physiological role in electrolyte homeostasis, regulation of systemic vascular resistance, and blood pressure [62, 112, 113]. The RAAS is known to function not only in kidney tissues but also in other organs (heart, lungs, liver, retina, etc.) [32, 112, 114]. The brain also has its own RAAS (cerebral RAAS), which, through autocrine, paracrine, and other intracerebral mechanisms, can influence various physiological and pathological cerebral processes independently of the systemic RAAS [115]. The component parts of the cerebral RAAS are synthesized locally within the brain [114]. For example, the precursor angiotensin I is primarily produced within astrocytes; renin is expressed within neurons and astrocytes; and angiotensin-converting enzyme is expressed in neurons, astrocytes, oligodendrocytes, and microglia of various brain sections [112, 116].

It is hypothesized that tissue angiotensin produced outside the brain may interact with cerebral RAAS receptors after entering the brain at sites lacking a BBB (the subfornical organ, medial areas of the corpus callosum, hippocampus, amygdala) or when its integrity is compromised [62, 112, 114, 117]. Some authors note that excessive activity of the cerebral RAAS has a direct impact on neuroglia. This effect is mediated via angiotensin II and the activation of various intracellular and extracellular pathways, which in turn leads to increased oxidative stress, neuroinflammation, apoptosis, and CND [112, 117]. Several clinical studies [62, 112, 118] report data on a direct connection between excessive activation of angiotensin receptors in the brain and CI. In support of this, there are reports of a positive effect of antihypertensive therapy with angiotensin II receptor blockers on cognitive functions [62, 112, 119].

Kang et al. [120] evaluated the 5-year survival of South Korean patients (n=54,903) receiving MHD and antihypertensive therapy (if needed). The highest survival rates were observed in patients who had not used antihypertensive drugs. Among those treated with antihypertensive medications, survival rates were higher in patients receiving RAAS blockers. The cognitive status of patients was not studied in this research.

It should be noted that convincing data on the influence of the cerebral RAAS on the development of CND and CI in MHD patients are currently lacking. The use of cerebral RAAS blockers, for example, with corresponding angiotensin II receptor antagonists, can also be considered promising for future clinical research aimed at neuroprotection and slowing the progression of CND and CI in this category of patients with concomitant arterial hypertension.

## Conclusion

CKD, whose outcome often requires MHD, is known to represent a model of accelerated aging. Therefore, understanding the mechanisms underlying the development of CND and CI in MHD patients is especially important. In the pathogenesis of CND and CI in MHD patients, specific attention is given to mechanisms and factors associated with neuroglial activation, namely: chronic systemic inflammation, persistent exposure to uremic toxins, and activation of the cerebral RAAS.

Potential science-based fields for future clinical research on this issue could include the development of strategies to reduce systemic inflammation and improve the cytokine profile of the “dialysis” patient, the use of S100B protein inhibitors crossing the BBB, as well as the use of uremic toxin adsorbents and cerebral RAAS inhibitors. Further research is needed to identify therapeutic targets, develop manageable modern strategies and technologies aimed at slowing the progression of CND and CI in MHD patients. The science-based development of these approaches will be possible as results from clinical studies accumulate, allowing for the establishment of clinical efficacy and safety for the proposed therapeutic strategies.
